# A38 INVESTIGATION OF POST-TRANSLATIONAL MODIFICATIONS IN SERUM OF CROHN’S DISEASE PATIENTS USING A PROTEOMICS APPROACH

**DOI:** 10.1093/jcag/gwac036.038

**Published:** 2023-03-07

**Authors:** L G N De Almeida, R Rosentreter, S Hirota, C Lu, A Dufour

**Affiliations:** 1 Department of Biochemistry and Molecular Biology; 2 Department of Medicine; 3 Department of Physiology and Pharmacology; 4 Department of Microbiology, Immunology & Infectious Diseases, University of Calgary, Calgary, Canada

## Abstract

**Background:**

Canada has one of the highest prevalences of Crohn’s disease (CD) worldwide. More specifically, fibrostenotic CD is a phenotype with prolonged chronic inflammation and fibrotic strictures often resistant to anti-inflammatory therapies and characterized by luminal narrowing that ultimately requires surgery. Proteins play an essential role in disease pathogenesis, and post-translational modifications (PTMs) can alter their properties. PTMs have been frequently implicated in human diseases. However, they have yet to be explored in the context of CD, which could lead to new avenues for a better understanding of disease mechanisms and the discovery of biomarkers.

**Purpose:**

Identify post-translational modifications in serum proteins of CD patients.

**Method:**

Serum samples from patients with strictures or inflammatory phenotype (without strictures) (n=4 per group), as diagnosed by intestinal ultrasound, were analyzed using a shotgun-proteomics approach. Protein identification and PTM prediction were performed with FragPipe. Identified mass shifts determined by an open search in FragPipe were mapped to possible PTMs and confirmed via unimod.org. Statistical significance analysis was performed with MSstatsPTM.

**Result(s):**

The prediction analysis identified 363 potential modification sites, including artifacts and chemical derivatives. The addition of all potential PTMs in the analysis would lead to false positives; therefore, it was selected five of the most abundant mass shifts mapped to true PTMs: cysteine oxidation, serine methylation, and three modifications of the protein n-termini (formaldehyde adduct, carbamylation, and formylation). Standard proteomics analysis identified 3635 unique peptides and 317 unique proteins. The addition of the predicted PTMs increased the number of peptides by 9.8%, reaching 3994 unique sequences. Interestingly, a very subtle increase was observed on the protein level, where only two additional proteins were identified. Of the PTMs identified, methylation of a serine residue on the variable chain of immunoglobulin (IGLV1-47) was statistically enriched in inflammatory samples (5.74 fold change, adj. p-value = 0.041). The variable chain participates in the antigen recognition process, and modification of its amino acids could impact antibody specificity. Additionally, structuring patients showed two modifications on thrombin: oxidation of cysteine and methylation of serine. Thrombin was previously shown to be elevated in CD patients compared to healthy controls. As both modifications were not present in inflammatory patients, they constitute potential biomarkers for specific diagnosis of the structuring disease.

**Conclusion(s):**

The observed gain in peptide identification demonstrates the diversification promoted by PTMs and exhibits their importance in proteomics studies. Even though the identified modifications require further validation, they can shed light on new players of CD pathogenesis and suggest novel biomarkers for disease diagnosis.

**Disclosure of Interest:**

None Declared

